# A bibliometric analysis of sleep in older adults

**DOI:** 10.3389/fpubh.2023.1055782

**Published:** 2023-02-23

**Authors:** Haitao Liu, Feiyue Liu, Haoyuan Ji, Zuanqin Dai, Wenxiu Han

**Affiliations:** ^1^College of Physical Education, Henan University, Kaifeng, Henan, China; ^2^Research Center of Sports Reform and Development, Henan University, Kaifeng, Henan, China; ^3^Institute of Physical Fitness and Health, Henan University, Kaifeng, Henan, China

**Keywords:** aged, older adults, sleep, bibliometrics, CiteSpace

## Abstract

**Background:**

Sleep problems severely affect the quality of life in the elderly and have gradually gained attention among scholars. As a major hot spot of current research, sleep in older adults is highly exploratory and of great significance for human health.

**Objective:**

Therefore, in this study, the current state of the art of sleep research in older adults was analyzed through the visual mapping function of CiteSpace software. Using this software, we analyzed popular research questions and directions and revealed the development trends and research frontiers of this field.

**Methods:**

In this paper, we searched the Web of Science database for sleep-related studies focusing on older adults and analyzed the number of publications, journals, authors, institutions, country regions, and keywords by using CiteSpace software.

**Results:**

Our results revealed that the number of publications concerning sleep in older adults has gradually increased; after 2017, this field underwent rapid development. The journal *Sleep* has published the majority of the articles on sleep in older adults and has the highest citation frequency. The *Journal of the American Geriatrics Society* has the highest impact factor and CiteScore among the top 10 journals in terms of the number of published articles. The United States has the highest number of publications and most of the leading institutions in this field are located in the United States, with the University of California, Los Angeles, and the University of Pittsburgh having the highest number of publications. Dzierzewski JM is the most published author and has played an important role in guiding the development of this field. Research in this area is focused on insomnia, sleep quality, depression, and sleep duration.

**Conclusion:**

The rapid development of sleep research in older adults, which shows a yearly growth trend, indicates that this field is receiving increasing attention from researchers. Insomnia in older adults is the most concerning problem in this field. At the same time, future research should continue to focus on the impact of sleep disorders on older adults to improve sleep and quality of life in older adults.

## 1. Introduction

The aging population experiences age-related symptoms, such as slowed metabolism, decreased resistance, and decreased physiological functions, and may even develop age spots and memory loss (1). With the increase in age, many older adults have difficulties in falling asleep, short sleep durations, and problems waking up too early, leading to insomnia, which severely affects sleep quality and the physical and mental health of older adults (2). An optimum sleep duration for older adults should be at least 7–8 h of undisturbed sleep daily (3). Due to factors such as age, sex, environment, physical illness, and psychology (4), the sleep patterns of older adults change with prolonged daytime sleep and insufficient nighttime sleep. Therefore, sleep patterns in older adults are easily disturbed by various factors, resulting in easy awakening at night, in addition to reduced sleep capacity and efficiency (5–7).

Sleep disorders (SDs) are symptoms of abnormal amounts of sleep, abnormal behaviors during sleep, and disruptions of the circadian rhythm (alternating patterns of sleep and wakefulness) (8, 9). SDs can be caused by a variety of factors, and they are closely linked to the health of a person. SDs are common in the older population, and the prevalence of SDs may increase due to aging, disease, medication, and psychological factors (10). SDs lead to several adverse consequences, such as memory loss, physical decline, cognitive impairment (11), and risk of falls (12). They can also make people prone to anxiety and depression, and can even increase the prevalence of mental illnesses (13). Therefore, SDs are often considered one of the important risk factor for death in older adults (13).

Good and sufficient sleep is beneficial to the recovery process from physical diseases, and it is necessary for undertaking daily activities. Furthermore, it can improve the quality of life in older adults (14), reduce negative emotions, and enhance physical health (15). There are various ways to improve sleep in the elderly, including exercise, diet, music, etc. Exercise is one of the most important ways. Studies have proven that physical activity can prolong sleep duration, improve heart rate, reduce psychological distress, and improve sleep quality, which are important factors in maintaining physical health in older adults (16). Regular exercise can improve sleep quality and sympathetic nerve function and reduce the risk of cardiovascular and metabolic diseases in older adults (17). Exercise promotes emotional health, reduces the psychological burden of older adults, improves sleep quality, and enhances the quality of life (18, 19). Dietary intake also affects sleep quality. One study found that foods rich in tryptophan and melatonin can improve sleep, and fruit and fiber intake are associated with sleep quality (20). Other studies have shown that music therapy is often used for emotional self-regulation, and insomnia is closely related to mood disorders. Physiologically, music can improve sleep quality in older adults through its effects on the endocrine and autonomic nervous systems (21).

Research on sleep in older adults has gradually increased in recent years. Since elderly sleep is influenced by many aspects, such as sex, sleep habits, physical and mental disorders, and medications, a considerable number of studies exist in this area, and their findings are complicated. Scientific knowledge mapping is a popular research method in the fields of scientometrics and visual analysis, which reveals the development patterns and research hot spots of related fields through visualization (22). With the help of bibliometric methods, we can systematically analyze the impact of sleep research to determine the current research trends and reveal the research dynamics in this area (23). CiteSpace is a citation visualization software program developed in the context of scientometrics and data visualization, which analyzes the citations in the literature of specific regions, presents the structure, pattern, and distribution of scientific knowledge through visualization, and transforms the visualized graphs obtained from the analysis into scientific knowledge maps (24). Therefore, in this paper, we used CiteSpace to analyze the current state of development and trends in sleep research involving older adults to explore popular research questions and directions, reveal the development dynamics and research frontiers in this field, and finally provide insights for its further advancement (25).

## 2. Data collection and research methods

### 2.1. Data collection

**Figure 1:**
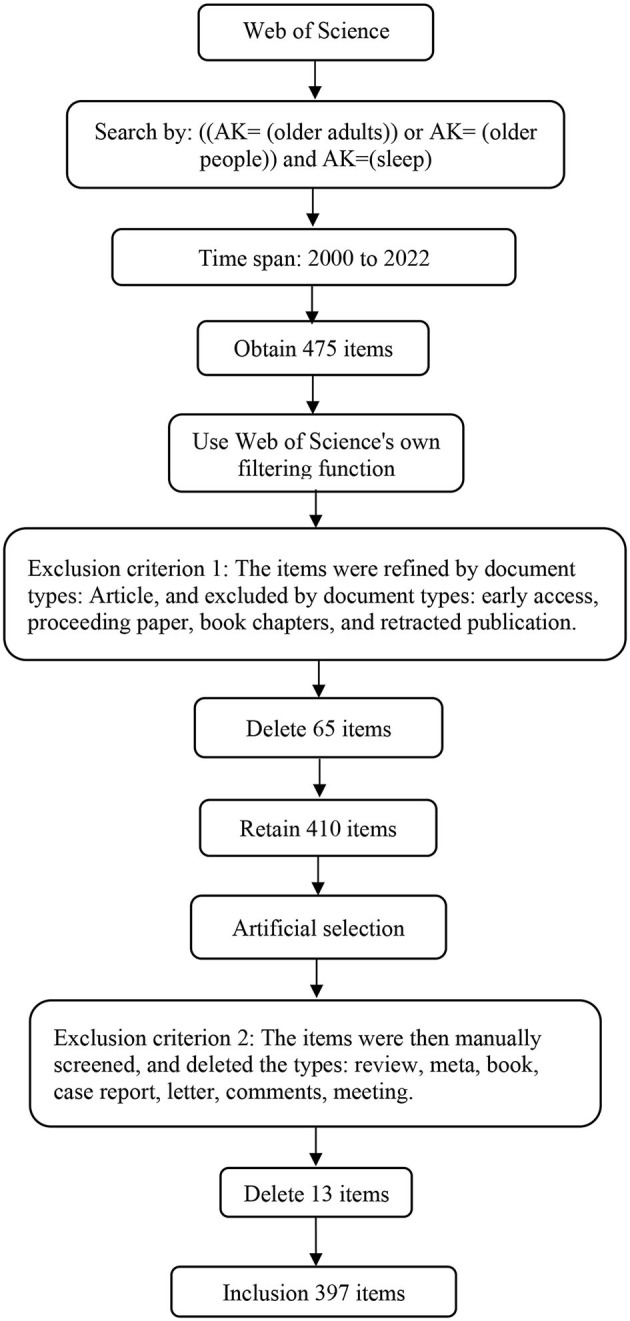
Database search flow chart.

The data used in this paper were obtained from the Web of Science (WOS) database from 1 January 2000 to 1 August 2022. The search was conducted using author keywords (AK) in the form [AK= (older adults) or AK= (older people)] and AK= (sleep). A total of 475 documents were retrieved, after which the items were refined by document type (article) and excluded by types of publication (early access, proceeding paper, book chapters, and retracted publication); thus, 65 items were excluded. The items were manually filtered by excluding reviews, meta, case reports, books, letters, comments, and meetings, through which 13 items were excluded. A total of 397 items were included in this study (Figure 1). The entire search and screening of the literature were performed on the same day. These retrieved studies were exported in plain text format with full records and cited references and imported into CiteSpace software. No duplicate records were found. The formatted data files in the “output” folder were copied and pasted into the “data” folder for subsequent data analysis.

### 2.2. Research methods

In this study, CiteSpace 6.1.R2 software was used to map scientific knowledge, and co-occurrence and clustering analyses were performed on high-frequency terms, research institutions, and countries. Finally, 397 papers were included in the study, and the trend maps of publication volume, authorship, institutional cooperation, geographical distribution, and keyword mapping were drawn to highlight key nodes and research hots pots, thus visualizing the field of sleep research in older adults. Each of these plots represents cutting-edge hot spots and trends in scientific research and can be selectively analyzed based on a particular aspect of the problem (24). In addition, when mapping, CiteSpace provides module values (Q-values) and average profile values (S-values) to ensure that mapping is effective. When Q> 0.3, the obtained network structure is significant, and when S> 0.5, the clustering is considered reasonable, whereas S> 0.7 indicates plausible clustering (24). CiteSpace can present domain-specific research trends through quantitative analysis and image visualization. It helps to reveal the development, hot spots, and frontiers of scientific research.

## 3. Results

### 3.1. Analysis of the volume of publication

**Figure 2:**
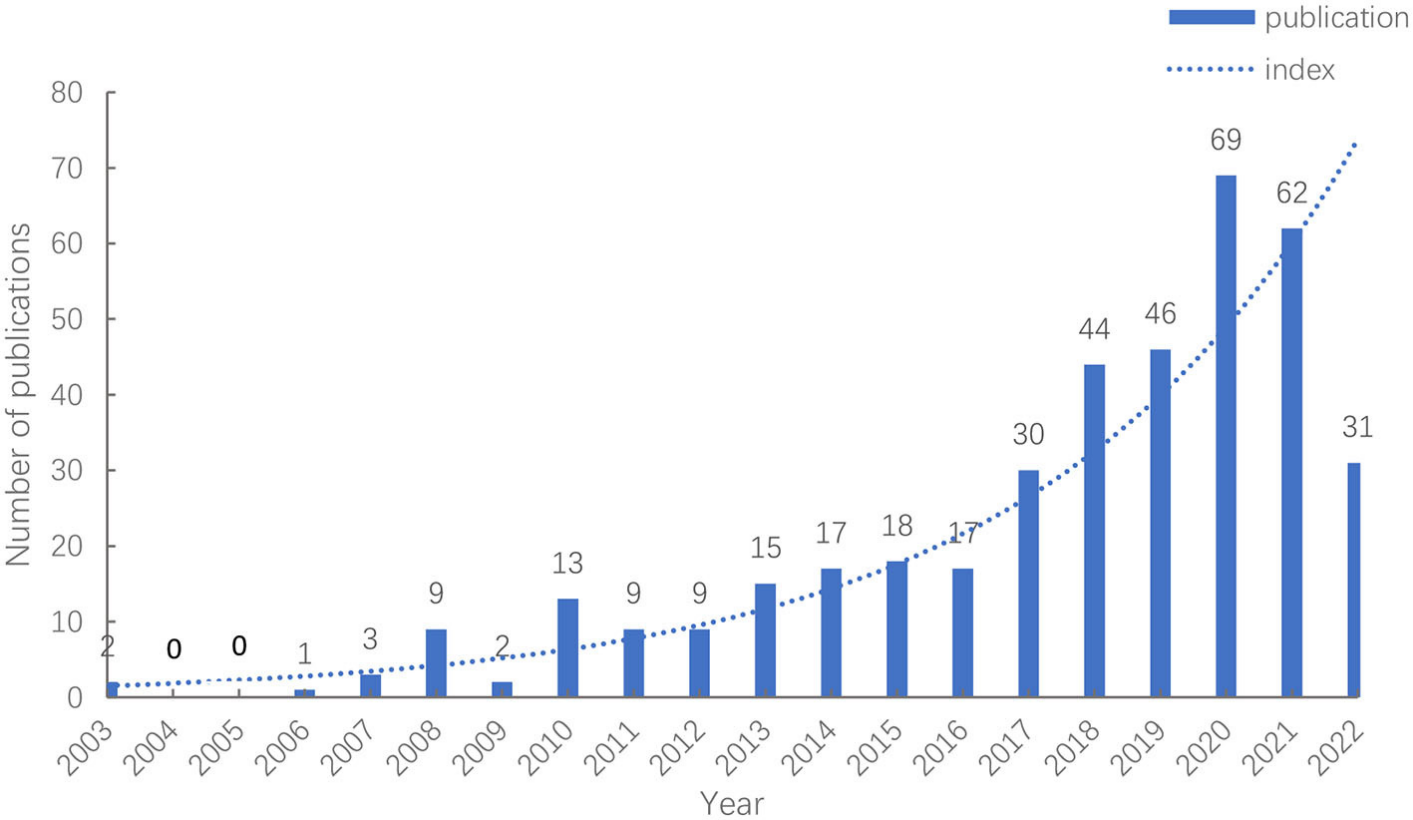
Publication trends over time.

After searching and screening, a total of 397 publications were found in the WOS database between 2000 and 2022. It can be seen in Figure 2 that the number of publications in the field of sleep research in older adults has gradually increased and more rapidly developed over time, showing a positive upward trend overall. In particular, the number of publications rapidly increased after 2017, with more than 60 articles published in 2020 and 2021.

### 3.2. Analysis of journals

The included literature was organized and ranked according to the number of published articles, resulting in the top 10 journals in terms of the number of articles (Table 1). The research literature focused on elderly sleep mainly involves journals related to sleep medicine, geriatrics, nursing, and psychology. The top 10 journals with the highest number of articles published a total of 134 articles, accounting for 33.8% of the total number of articles. Among them, *Sleep* ranks first, with 23 articles, and is also first in terms of citation frequency (508). Ranking second and third are the *Journal of Clinical Sleep Medicine* and *Sleep Medicine*, with 22 and 19 articles, respectively. The *Journal of the American Geriatrics Society* ranks fourth in terms of the number of published articles. This journal ranks first in both impact factor (7.538) and CiteScore (8.8). Overall, 5 of the top 10 journals in terms of the number of published articles are from the United States. This is followed by the United Kingdom, with 4 of the top 10 journals.

**Table 1:** Top 10 journals by publications.

**Rank**	**Periodicals**	**Publications**	**Citations**	**Country**	**Category zone**	**IF (2021)**	**CiteScore**
1	*Sleep*	23	508	United States	Q1	6.313	8.0
2	*Journal of Clinical Sleep Medicine*	22	268	United States	Q2	4.342	5.7
3	*Sleep Medicine*	19	319	Netherlands	Q2	4.842	5.2
4	*Journal of the American Geriatrics Society*	14	489	United States	Q1	7.538	8.8
5	*International Journal of Environmental Research and Public Health*	10	47	Switzerland	Q1	4.614	4.5
6	*BMC Geriatrics*	9	74	United Kingdom	Q2	4.070	4.8
7	*Aging & Mental Health*	8	96	United Kingdom	Q2	3.514	5.5
7	*Geriatric Nursing*	8	46	United States	Q2	2.525	2.4
8	*Clinical Gerontologist*	7	54	United Kingdom	Q2	2.871	5.1
8	*International Psychogeriatrics*	7	102	United States	Q1	7.191	7.1
8	*International Journal of Geriatric Psychiatry*	7	58	United Kingdom	Q2	3.850	6.3

### 3.3. Analysis of literature

The top 10 most frequently cited articles are listed in Table 2. The most frequently cited article is *Effects of moderate-intensity exercise on polysomnographic and subjective sleep quality in older adults with mild to moderate sleep complaints*. This article has 146 citations and is authored by King AC. The article was published in 2008. Among the top 10 cited articles, 4 articles were published in 2010 in *Sleep Medicine, Sleep, Psychoneuroendocrinology*, and *Journal of Medicinal Food*. Most of the highly cited articles were published in American journals, namely *Journals of Gerontology Series A-Biological Sciences and Medical Sciences, Sleep, American Journal of Geriatric Psychiatry*, and *Medical Clinics of North*.

**Table 2:** Top 10 papers with the most citations.

**Rank**	**Title**	**Author**	**Periodicals**	**Journal publishers**	**Country**	**Frequency of citations**	**Year**
1	Effects of moderate-intensity exercise on polysomnographic and subjective sleep quality in older adults with mild to moderate sleep complaints	King AC	*Journals of Gerontology Series A-Biological Sciences and Medical Sciences*	Oxford University Press Inc	United States	146	2008
2	Sleep-disordered breathing and cognition in older women	Spira AP	*Journal of the American Geriatrics Society*	Wiley	United States	117	2008
3	Daytime sleepiness is associated with dementia and cognitive decline in older Italian adults: a population-based study	Merlino G	*Sleep Medicine*	Elsevier	Netherlands	105	2010
4	Sociodemographic and health correlates of sleep quality and duration among very old Chinese	Gu DN	*Sleep*	Oxford University Press Inc	United States	102	2010
5	A change in sleep pattern may predict Alzheimer's disease	Hahn EA	*American Journal of Geriatric Psychiatry*	Elsevier Science Inc	United States	98	2014
6	Sleep disturbance in relation to health-related quality of life in adults: the Fels Longitudinal Study	Lee M	*Journal of Nutrition Health & Aging*	Springer France	France	90	2009
7	The effect of a simple traditional exercise programme (Baduanjin exercise) on sleep quality of older adults: a randomized controlled trial	Chen MC	*International Journal of Nursing Studies*	Pergamon-Elsevier Science Ltd	United Kingdom	80	2012
8	Afternoon napping and cognition in Chinese older adults: findings from the China health and retirement longitudinal study baseline assessment	Li JX	*Journal of the American Geriatrics Society*	Wiley	United States	79	2017
9	What constitutes too long of a delay? Determining the cortisol awakening response (CAR) using self-report and PSG-assessed wake time	Okun ML	*Psychoneuroendocrinology*	Pergamon-Elsevier Science Ltd	United Kingdom	74	2010
10	Effects of a tart cherry juice beverage on the sleep of older adults with insomnia: a pilot study	Pigeon WR	*Journal of Medicinal Food*	Mary Ann Liebert Inc	Korea, South	73	2010
10	Sleep problems in the elderly	Rodriguez JC	*Medical Clinics of North America*	WB Saunders Co-Elsevier Inc	United States	73	2015

### 3.4. Analysis of country/region

**Figure 3:**
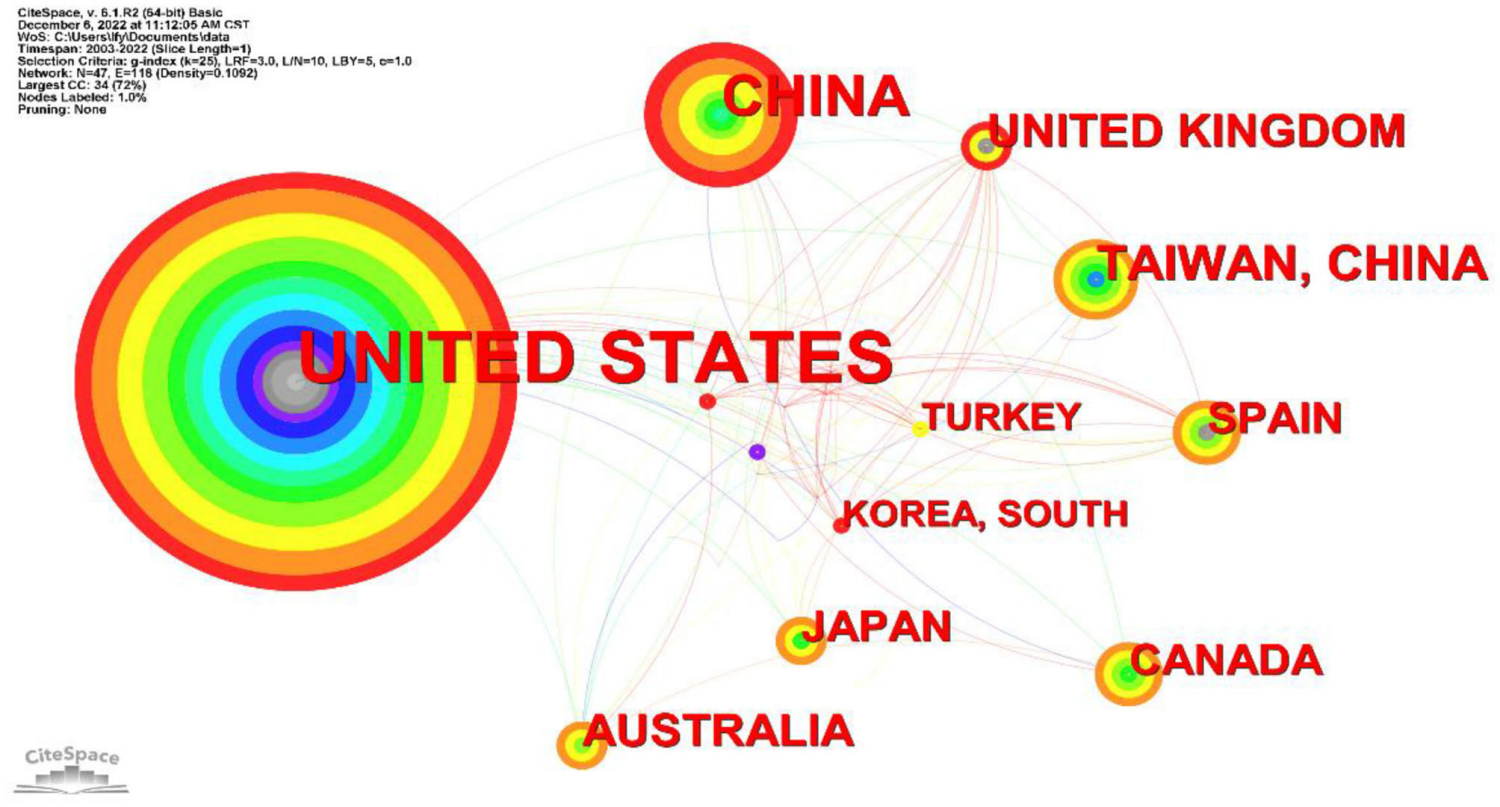
Country/region collaboration chart.

As seen from Table 3, the United States is the most active in the field of sleep research in older adults, with the highest number of published papers, accounting for 177 papers (44.6%) of the total. China ranks second with 57 papers, accounting for 14.4% of the total. Taiwan, China, is third with 30 articles, followed by the United Kingdom and Australia with 21 articles each. Canada, Japan, and Spain have 18 articles each, 12 articles have been published in Turkey and 11 articles in Korea, South. Figure 3 is an alternative representation of country/region by the number of publications. The regional distribution of countries can reveal the level of academic research collaboration among various country/region. As shown in Figure 3, there are 47 nodes with 118 connected lines and a network density of 0.1092. This figure shows that researchers in many countries are conducting studies on sleep quality in older adults, with the United States and China forming a relatively dense network of collaboration.

**Table 3:** Top 10 country/region by the number of publications.

**Rank**	**Country/region**	**Number of publications**	**Percentage (%)**
1	United States	177	44.6
2	China	57	14.4
3	Taiwan, China	30	7.6
4	Australia	21	5.3
4	United Kingdom	21	5.3
5	Canada	18	4.5
5	Japan	18	4.5
5	Spain	18	4.5
6	Turkey	12	3.0
7	Korea, South	11	2.6

### 3.5. Analysis of institutional and author collaboration

**Figure 4:**
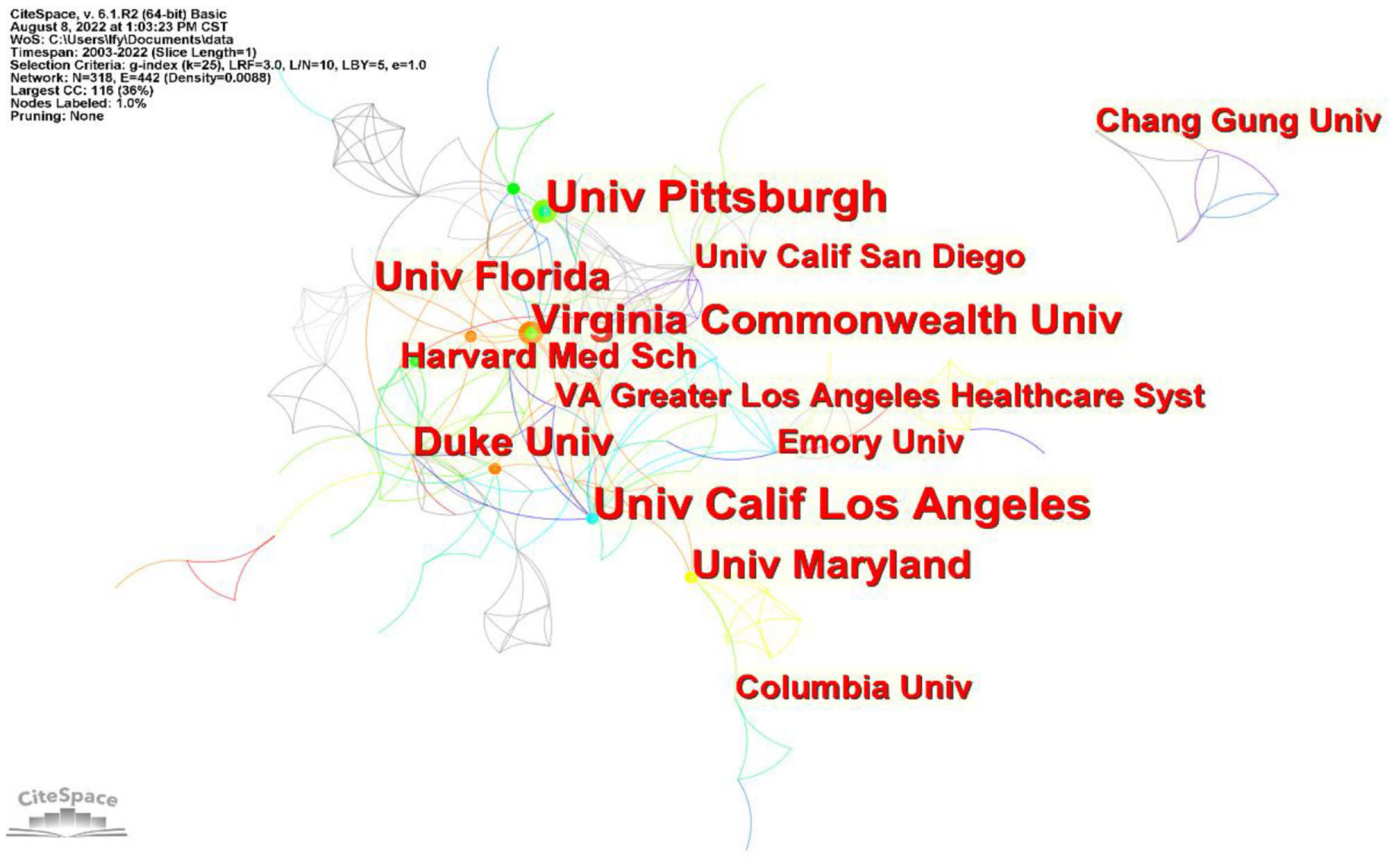
Institution collaboration chart.

Figure 4 illustrates the collaboration among institutions in the field of sleep research in older adults. Figure 4 and Table 4 show that the institutions with more publications are mainly universities, with the most publications originating from the University of California, Los Angeles, and the University of Pittsburgh. Both universities are tied for first place with 14 publications. Some other influential research institutions include Virginia Commonwealth University, Duck University, University of Maryland, University of Florida, etc. All the top 10 institutions are from the United States, except Chang Gung University, which is located in Taiwan, China.

**Table 4:** Top 10 institutions in terms of number of articles issued.

**Rank**	**Country/region**	**Institutions**	**Number of publications**
1	United States	University of California, Los Angeles	14
1	United States	University of Pittsburgh	14
2	United States	Virginia Commonwealth University	11
3	United States	Duck University	10
3	United States	University of Maryland	10
3	United States	University of Florida	10
4	United States	Harvard Medical School	7
5	Taiwan, China	Chang Gung University	6
5	United States	Columbia University	6
5	United States	Emory University	6
5	United States	University of California, San Diego	6
5	United States	VA Greater Los Angeles Healthcare System	6

**Figure 5:**
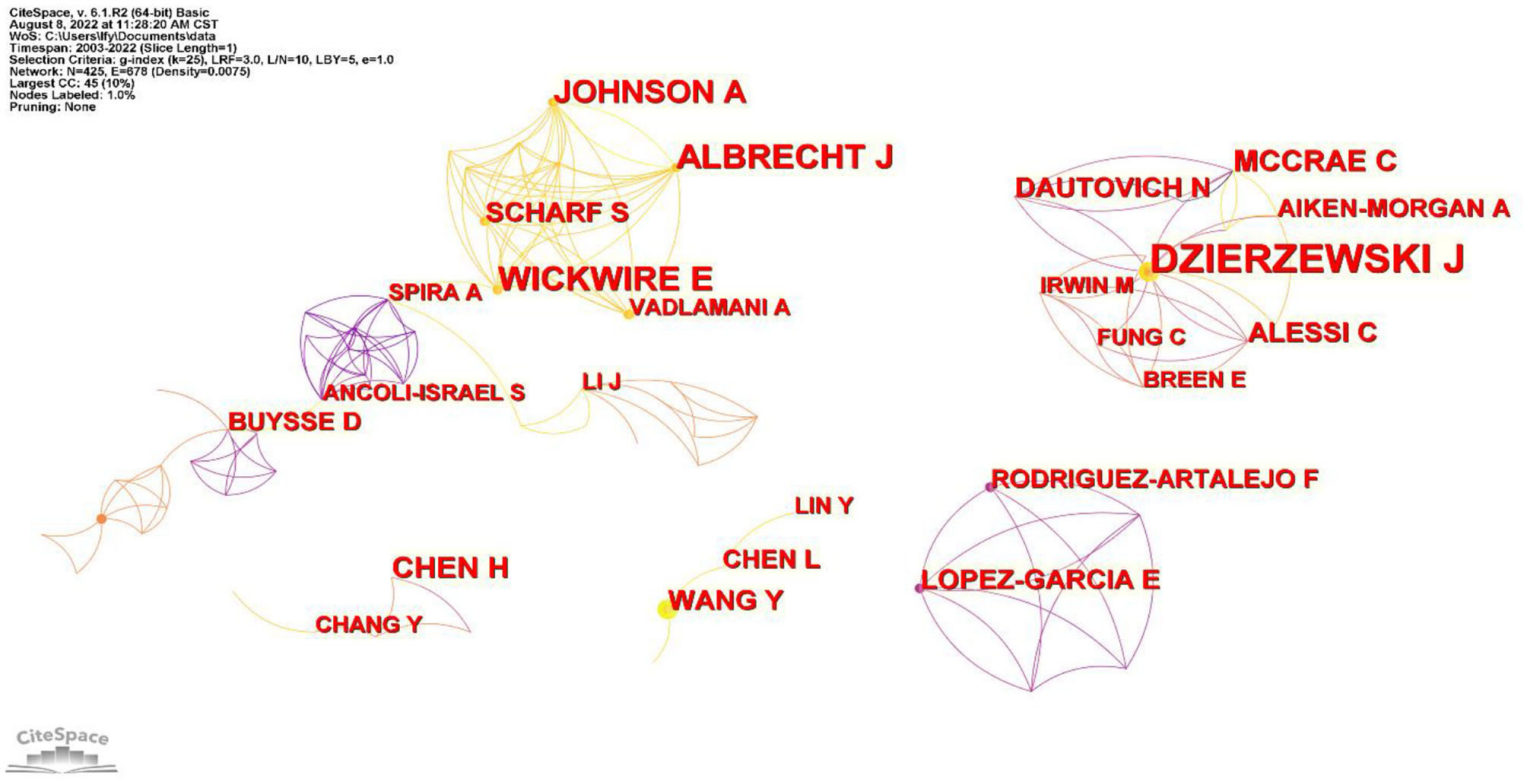
Author collaboration chart.

The number and size of the nodes in the author collaboration network map show the frequency of co-occurrence, and the number of connected lines indicates the collaborative network between authors, as well as its strength (24). There are 425 nodes and 678 connected lines in Figure 5, with a network density of 0.0075. Table 5 reveals that authors from the United States are highly productive and the most cited. The 4 authors with 10 or more publications are Dzierzewski JM, Mccrae CS, Wickwire EM, and Albrecht JS. Dzierzewski JM from Virginia Commonwealth University, United States, ranks first with 18 publications and has the highest citation frequency (353). Mccrae CS from the United States and Chen HC from Taiwan, China, with 287 and 165 citations, rank second and third, respectively (Table 5).

**Table 5:** Top 10 authors by number of publications.

**Rank**	**Author**	**Publications**	**Affiliation**	**Country/region**	**Total citations**	**Average citations**
1	Dzierzewski JM	18	Virginia Commonwealth University	United States	353	19.61
2	Mccrae CS	14	University of Missouri System	United States	287	20.5
3	Wickwire EM	11	University of Maryland Baltimore	United States	106	9.64
4	Albrecht JS	10	University of Maryland Baltimore	United States	101	10.10
5	Johnson AM	9	University of Maryland Baltimore	United States	51	5.67
5	Scharf SM	9	University of Maryland Baltimore	United States	94	10.44
6	Dautovich ND	8	Virginia Commonwealth University	United States	151	18.88
7	Chen HC	7	National Taiwan University	Taiwan, China	165	23.57
8	Alessi CA	6	VA Greater Los Angeles Healthcare System	United States	106	17.67
8	Wang Y	6	Chongqing Medical University	China	3	0.50

### 3.6. Keyword analysis

#### 3.6.1. Keyword co-occurrence analysis

**Figure 6:**
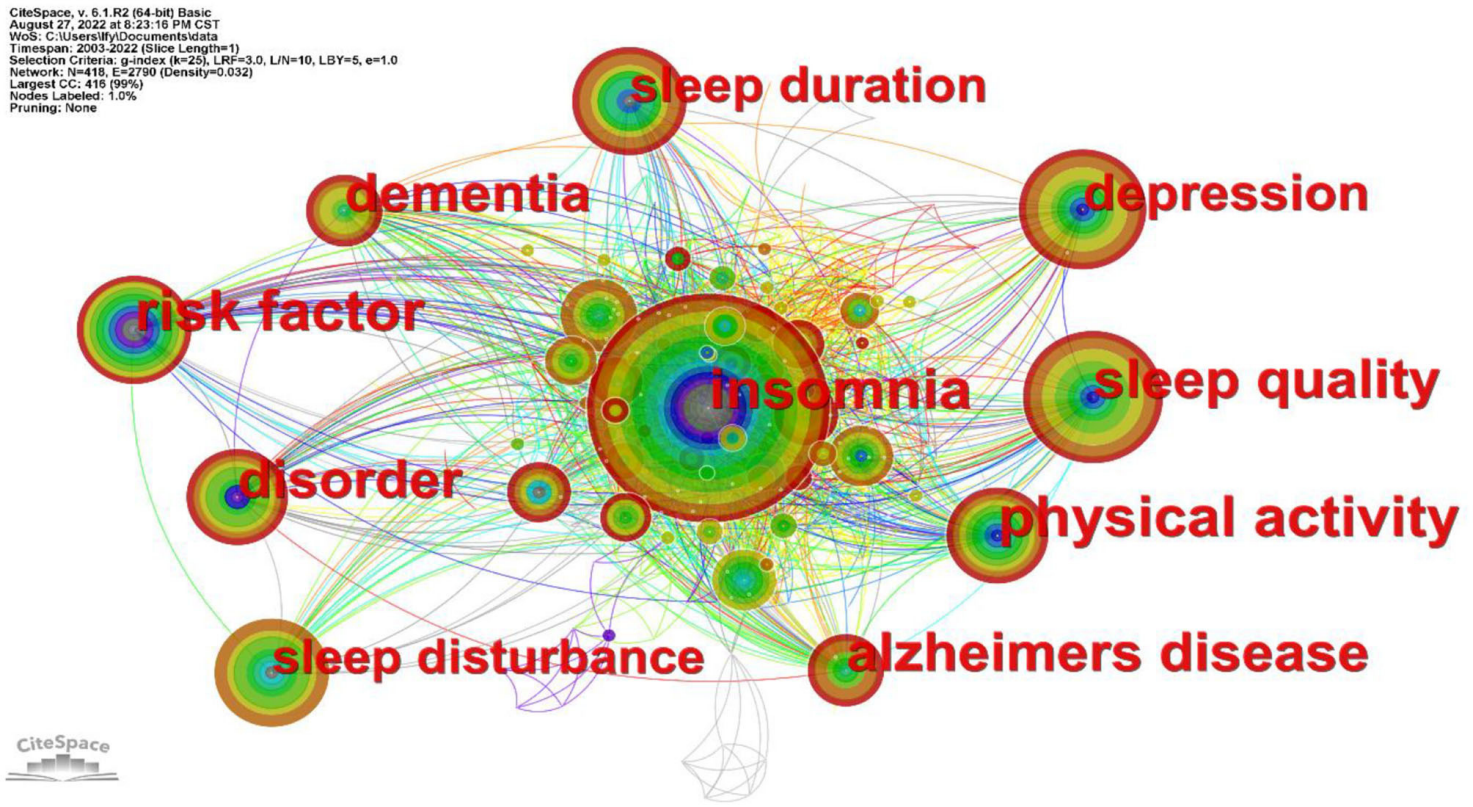
Keyword co-occurrence knowledge map.

The visual analysis of sleep in older adults was performed using CiteSpace software (Figure 6), and the time slice was set to 1 year, generating 418 nodes with 2,790 connections and a network density of 0.032. The top 10 keywords in terms of frequency of occurrence are listed in Table 6. The most frequent keywords are “insomnia”, “sleep quality”, “depression”, “sleep duration”, “disorder”, etc.

**Table 6:** Top 10 keywords co-occurrence frequency.

**Rank**	**Keywords**	**Number of occurrences**	**Centrality**
1	Insomnia	91	0.10
2	Sleep quality	73	0.08
3	Depression	49	0.05
4	Sleep duration	47	0.02
5	Disorder	45	0.04
6	Physical activity	41	0.12
7	Risk factor	40	0.09
8	Dementia	39	0.06
9	Sleep disturbance	37	0.03
10	Alzheimer's disease	32	0.06

#### 3.6.2. Keyword clustering analysis

**Figure 7:**
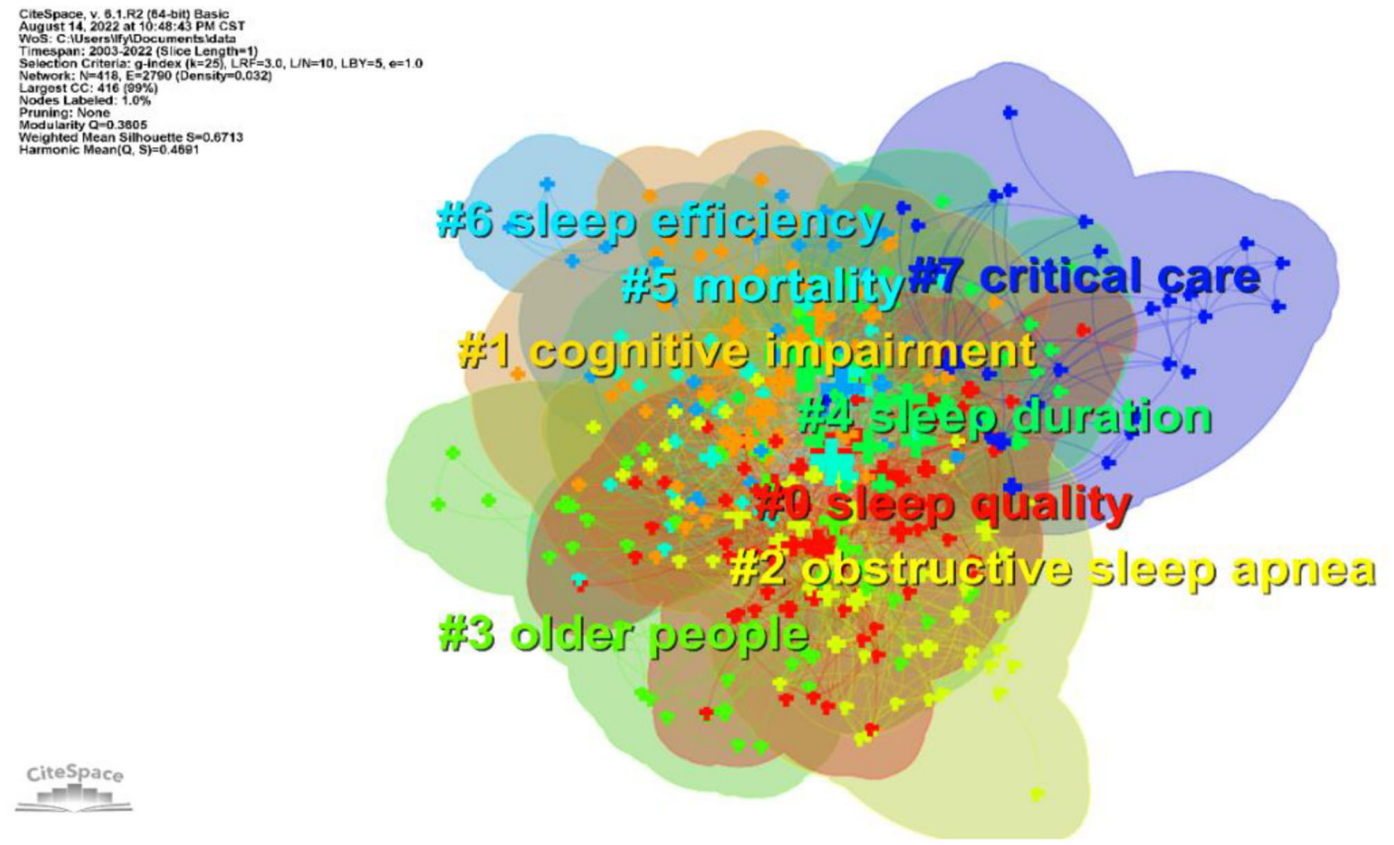
Keyword clustering and time zone visual mapping.

In order to better understand the research hot spots in the field of sleep in older adults, a keyword clustering map was drawn by running the CiteSpace visualization software (Figure 7). The scientific nature of the clustering map can be mainly inferred from the module value (Q value) and the average profile value (S value) (22). In this study, we obtained Q = 0.3605, which indicates a good clustering effect, and S = 0.6713, which indicates high homogeneity among the clusters and reasonable clustering results. Additionally, some mapping clusters overlap with each other, indicating that they are closely related. According to the clustering atlas listing and the relevant information of clusters (Table 7), the larger clusters include #0 sleep quality, #1 cognitive impairment, #2 obstructive sleep apnea, #3 older people, #4 sleep duration, #5 mortality, #6 sleep efficiency, and #7 critical care.

**Table 7:** List of keyword clusters.

**Rank**	**Size**	**Silhouette**	**Cluster**	**Keywords**
#0	72	0.553	Sleep quality	Sleep quality; self-perceived health status; primary care; sleep onset; physical activity
#1	61	0.559	Cognitive impairment	Sleep quality; cognitive function; subjective memory complaints; sleep disturbance
#2	52	0.629	Obstructive sleep apnea	Sleep-disordered breathing; sleep disturbance; sleep disorders; sleep assessment
#3	46	0.752	Older people	Sleep duration; sleep quality; sedentary lifestyle; medical disease
#4	35	0.752	Sleep duration	Sleep difficulty; health economics; sleep quality; older people; all-cause mortality
#5	33	0.776	Mortality	Sleep; older adults; emotions; clinical management; pain
#6	30	0.72	Sleep efficiency	Sleep quality; sleep efficiency; sleep deprivation; risk factors
#7	30	0.738	Critical care	Older adults; sleep; aging; critical care

**Figure 8:**
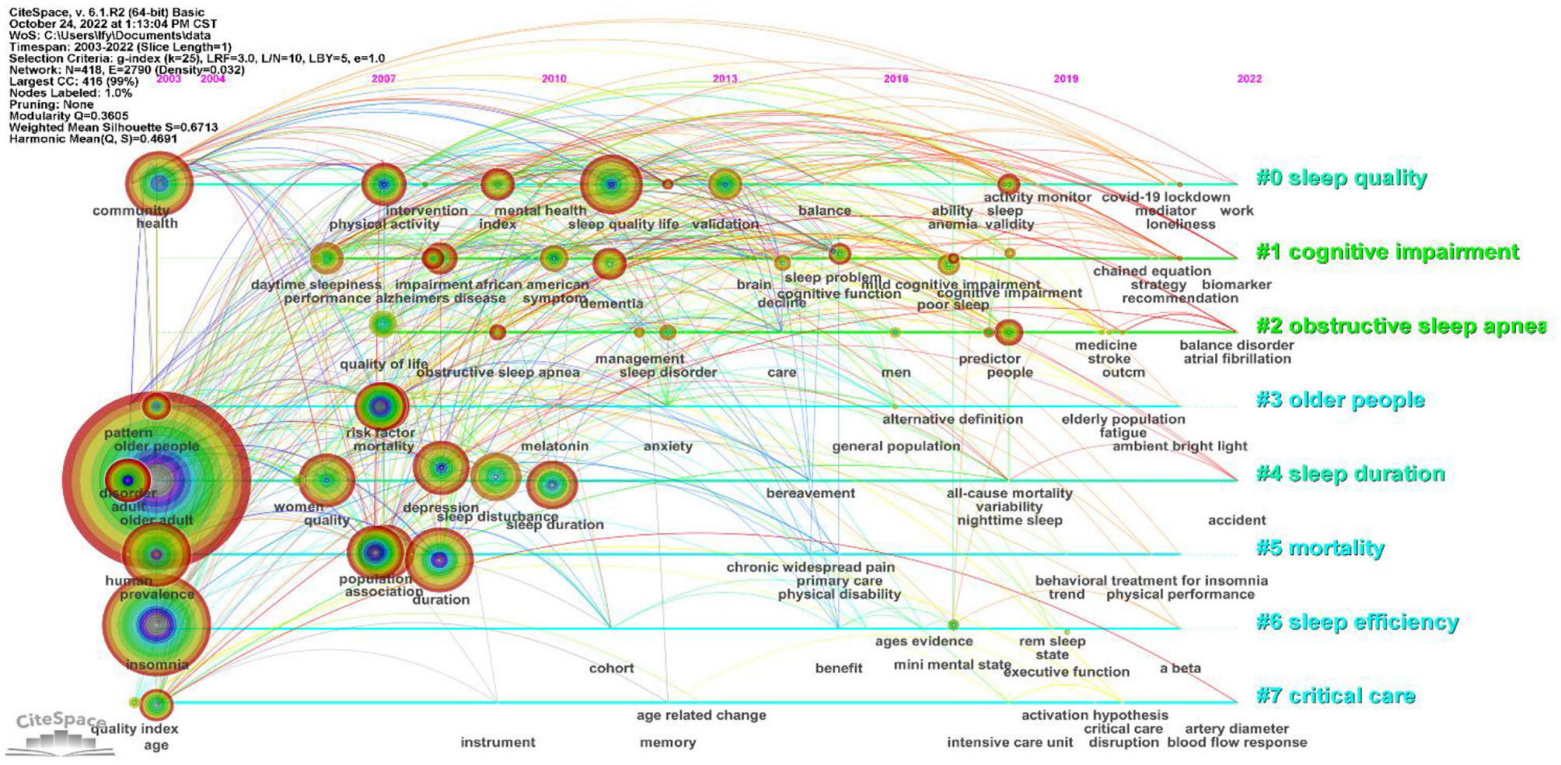
Keyword clustering and visual time-zone mapping.

To better visualize the research dynamics of sleep research in older adults, a keyword time-zone mapping was developed to show the evolution of research hot spots from 2003 to 2022 (Figure 8). High-frequency keywords mainly revolve around “sleep quality”, “older people”, “sleep duration ”, “mortality”, “sleep efficiency”, and “critical care”. The first series of high-frequency keywords appeared in 2003, including “health”, “older adults”, “insomnia”, “prevalence”, etc. Next, in 2007, other keywords emerged such as “physical activity”, “quality of life”, “risk factor”, “mortality”, “population”, “association”, etc. From 2007 to 2011, “duration”, “depression”, “sleep quality”, etc., increasingly appeared. From 2012 to 2022, other keywords emerged one after another. Based on the keyword analysis in Table 7, it is clear that the popular research topics involving elderly sleep are mainly found in the areas of sleep quality, cognitive dysfunction, obstructive sleep apnea, sleep duration, sleep efficiency, and emergency care.

## 4. Discussion

With aging-related physiological changes, the sleep schedule of the elderly also changes, with shorter nighttime sleep, more frequent daytime sleep, and reduced sleep duration. The underlying causes for these problems include physical diseases, mental problems, environment, and living conditions. Through the bibliometric analysis of research on elderly sleep with CiteSpace, the current situation and development trend of the elderly sleep research were identified in terms of authors, institutions, journals, countries, and keywords.

The trends in sleep research on older adults are reflected by the number of published papers in recent years. Figure 2 indicates that sleep research in older adults has continuously received extensive attention from scholars in recent years. This is caused by a rapid increase in the aging population in various countries and societies around the world. The incidence of SDs in older adults is increasing due to more people living alone, aging, and declining health (12). As the availability of the literature in this field continues to increase, a more in-depth understanding of sleep patterns in older adults will simultaneously develop.

*Sleep* ranks first in terms of the number of articles. This indicates that the journal has a high academic advantage and influence in the study of sleep in older adults. The main research directions of *Sleep* are behavioral sciences, clinical neurology, neurosciences, and psychiatry. The top three journals are all in the field of clinical neurology. The *Journal of the American Geriatrics Society* focuses on geriatrics gerontology. The journal is ranked first in both impact factor and CiteScore, indicating the high quality of academic papers published in the journal. Other journals in the field of geriatrics include *BMC Geriatrics, Geriatrics Nursing, International Psychogeriatrics, International Journal of Geriatric Psychiatry*, and *Clinical Gerontologist*.

Highly cited research is an essential source of scientific knowledge in any field of study, as it indicates the level of research and trends of development in that field and is the scientific basis for exploring the frontiers of research and hot spots (24). The citation frequency of the literature is an important indicator of the influence and quality evaluation of papers, and it is used to evaluate the state of the influence and the quality level of scientific research in different countries, institutions, or individuals (22). The most frequently cited article is *Effects of moderate-intensity exercise on polysomnographic and subjective sleep quality in older adults with mild to moderate sleep complaints*. The article focused on the effects of exercise interventions on sleep in older adults. It revealed that moderate-intensity exercise improved sleep quality in older adults with mild-to-moderate sleep complaints (26).

The country–region distribution map is a much better depiction of the scientific research differences and distribution in sleep research in each country. Far more papers are published in the United States than in other countries. This indicates that the United States has the highest level of research and influence in the field of sleep research. Figure 3 shows that sleep-related articles surfaced in the United States earlier than in other countries and that the number of published articles is relatively stable. Our results also show that the United States engages in collaborative exchanges with most countries. In contrast, China, Australia, Canada, and other countries have published more articles in recent years. Academic exchanges between various countries and regions are more frequent and closer, which makes the development of sleep research in older adults a positive and continuous process.

University of California, Los Angeles, and the University of Pittsburgh rank first in terms of the number of published papers, indicating their outstanding contributions to this field. The University of California, Los Angeles is the cradle of scientific research and high-tech industry professionals in the United States. It invests a considerable amount of human and material resources into research every year. The mapping of institutional collaboration shows that several US research institutions have active collaborative networks with other institutions and that more opportunities exist for academic exchange and learning. Various institutions learn from each other and contribute to the continued development of related research.

Author collaboration mapping provides a clear view of the research status of individual scholars in a research area and the collaboration among them (Figure 5). It provides a visual representation of the more prolific authors. The study of highly productive authors is more helpful to grasp new trends in elderly sleep research. Additionally, the scholarly influence of authors can be assessed by the number of publications and the frequency of citations, which reflect the academic ability of authors and the recognition of scholars (27). Sleep research in older adults reveals close cooperation among researchers and opportunities for mutual learning and communication. This collaboration is conducive to in-depth advances in this field and has a positive impact on the future direction of its development.

Keywords are high-level summaries of the topic of a paper and reflect the research direction and value of the article. Analysis of keywords in the related literature of a certain field helps explore the research hotspots and development trends in that field (28). Keyword co-occurrence refers to the appearance of different keywords in the same literature (23). A high number of keyword occurrences reflects research hot spots and the developmental directions of the field (29). This analysis indicated that the topics of sleep-related research in older adults have been more concentrated and closely connected in recent years. From keyword analysis, the main condition affecting elderly sleep was found to be insomnia. Insomnia is one of the most common SDs in older adults, and it mainly manifests as difficulty falling asleep and maintaining sleep (30). Divorce, widowhood, smoking, drinking, infrequent participation in sports, and other factors also lead to a higher rate of insomnia in the elderly (31). Older adults with sleep problems are more likely to develop mental disorders such as anxiety and depression (32). Long-term poor sleep is the main etiology for the development of depressive symptoms in older adults. Depressed adults with SDs may experience more severe symptoms, which makes their treatment difficult (33). Keeping the body and mind active and in a good emotional state is an effective strategy against depression. Chronic insomnia in older adults may lead to cognitive dysfunction, causing neuronal damage and memory loss (34). This can increase the risk of dementia and Alzheimer's disease in older adults (35). The risk of insomnia is higher in older adults with respiratory illnesses, and physical disabilities, who need to take medications more often than older adults in better health (36). Other factors that contribute to SDs are behavioral, such as the use of electronic devices at bedtime, which suppresses melatonin production and leads to circadian rhythm disturbances (37). Environmental factors, such as room temperature, light intensity (38), and sound, can affect sleep (39). Poor nutritional conditions can also lead to reduced sleep quality in older adults (40). However, dietary habits can improve sleep in older adults. Increasing the daily consumption of fruits, meat, and eggs can increase the duration of sleep (41). Physiological factors such as snoring, apnea, and abnormal sleep behavior and duration can also interfere with sleep (42). Studies have found that improving physical activity levels has a positive impact on sleep in older adults (43). Therefore, regular exercise will also improve sleep quality, increase sleep duration, prevent cognitive decline, and improve the quality of life of older adults (44).

Some studies have shown that approximately half of older adults suffer from SDs, which is closely related to physical health status (45). The duration of sleep in older adults gradually decreases with age, as the total sleep time is shortened, sleep duration is reduced, sleep efficiency is lowered, waking time at night is prolonged, drowsiness during the day increases, and the circadian rhythm of the body is impaired (46, 47). It was found that the duration of sleep in older adults is related to the degree of frailty; thus, fit older adults sleep better. Additionally, it was observed that sleep is correlated with sex (48). Poor sleep quality and reduced sleep duration are signs of cognitive decline in older adults and can lead to memory loss and depressive symptoms (49). Furthermore, excessive sleep duration can impair cognitive and physiological function in older adults (50). Curtis et al. found that the association between self-reported sleep and cognitive performance interacted with pain in sedentary adults aged 50 years and above (51). Older adults have less energy, spend less time being physically active, and often maintain a sedentary lifestyle, which can lead to a decline in physical health (52). Therefore, older adults should perform more physical activities to improve their sleep quality and reduce sleep disorders. Exercise is an important measure to achieve physical and mental relaxation (53).

## 5. Limitations

This bibliometric analysis of studies involving elderly sleep was conducted with the help of CiteSpace software, using visual mapping to more graphically reveal the trends and patterns in this field, which in turn led to a series of new findings and interpretations. This paper focused on the number of publications, journals, authors, institutions, countries, and keywords, aiming at a macro analysis of the field, so some important detailed parts were not reflected in the study. CiteSpace software was developed based on the Web of Science Core Collection and, therefore, generally relies on this database for analysis. Thus, our choice of databases was limited. Additionally, we limited the search period to January 2000–August 2022, and those articles published after this period were not included.

## 6. Conclusion

This bibliometric study focused on sleep quality in older adults, providing detailed information on the relevant literature from a global perspective through visualization with CiteSpace. We found that the number of studies in the field of elderly sleep has increased year by year, especially after 2017, with a sharp increase in the number of publications, indicating that this research area has excellent growth potential. Pioneering research on elderly sleep originates from the United States. The research institutions involved in this area are mainly universities. Research hot spots revolve around insomnia, sleep quality, depression, sleep duration, etc. Research topics include sleep quality, cognitive impairment, obstructive sleep apnea, etc. To summarize, this study predicts future research trends in elderly sleep through visual analysis and can provide valuable insight to help understand the research frontiers and hot spots in this field. At the same time, sleep research in older adults is highly exploratory and deserves further development.

## Data availability statement

The original contributions presented in the study are included in the article/supplementary material, further inquiries can be directed to the corresponding author.

## Author contributions

HL contributed to conceptualization, design, and supervision. HJ and WH collected data from the database. FL and ZD analyzed the data. HL and FL wrote the original draft. HL made critical revisions of the manuscript. All authors have read and agreed to the published version of the manuscript.
